# Resignation of Professor Hamilton, of Geneva

**Published:** 1848-08

**Authors:** 


*Resignation of Professor Hamilton, of Geneva.*—Prof. F. H Hamilton has resigned his professorship at Geneva Medical College, and will hereafter be connected with the Medical Department of the University of Buffalo, exclusively. Dr. Ryan, of Philadelphia, has been appointed to the chair of Principles and Practice of Surgery, made vacant by the resignation of Dr. Hamilton.

